# Outcome measure for the treatment of cone photoreceptor diseases: orientation to a scene with cone-only contrast

**DOI:** 10.1186/s12886-015-0085-0

**Published:** 2015-08-08

**Authors:** Alejandro J. Roman, Artur V. Cideciyan, Rodrigo Matsui, Rebecca Sheplock, Sharon B. Schwartz, Samuel G. Jacobson

**Affiliations:** Scheie Eye Institute, Perelman School of Medicine, University of Pennsylvania, 51 North 39th St., Philadelphia, PA 19104 USA

**Keywords:** Inherited retinal degenerations, Achromatopsia, Cone dystrophy, Cone-rod dystrophy, Blue-cone monochromacy, Leber congenital amaurosis, Orientation and mobility, Treatment trials, Outcome measures, Low vision

## Abstract

**Background:**

Inherited retinal degenerations (IRDs) preferentially affecting cone photoreceptor function are being considered for treatment trials aiming to improve day vision. The purpose of the current work was to develop cone-specific visual orientation outcomes that can differentiate day vision improvement in the presence of retained night vision.

**Methods:**

A lighted wall (1.4 m wide, 2 m high) resembling a beaded curtain was formed with 900 individually addressable red, blue and green LED triplets placed in 15 vertical strips hanging 0.1 m apart. Under computer control, different combination of colors and intensities were used to produce the appearance of a door on the wall. Scotopically-matched trials were designed to be perceptible to the cone-, but not rod-, photoreceptor based visual systems. Unmatched control trials were interleaved at each luminance level to determine the existence of any vision available for orientation. Testing started with dark-adapted eyes and a scene luminance attenuated 8 log units from the maximum attainable, and continued with progressively increasing levels of luminance. Testing was performed with a three-alternative forced choice method in healthy subjects and patients with Leber congenital amaurosis (LCA) caused by mutations in *GUCY2D*, the gene that encodes retinal guanylate cyclase-1.

**Results:**

Normal subjects could perform the orientation task using cone vision at 5 log attenuation and brighter luminance levels. Most *GUCY2D*-LCA patients failed to perform the orientation task with scotopically-matched test trials at any luminance level even though they were able to perform correctly with unmatched control trials. These results were consistent with a lack of cone system vision and use of the rod system under ambient conditions normally associated with cone system activity. Two *GUCY2D*-LCA patients demonstrated remnant cone vision but at a luminance level 2 log brighter than normal.

**Conclusions:**

The newly developed device can probe the existence or emergence of cone-based vision in patients for an orientation task involving the identification of a door on the wall under free-viewing conditions. This key advance represents progress toward developing an appropriate outcome measure for a clinical trial to treat currently incurable eye diseases severely affecting cone vision despite retained rod vision.

## Background

Inherited retinal degenerations (IRD) are a molecularly heterogenous group of diseases that cause vision loss [1]. IRDs remain currently incurable but preclinical studies have been generating dozens of potential treatment avenues which are slowly moving to clinical trials [2]. The ultimate proof of value of any treatment must come from rigorously-conducted clinical trials that have chosen appropriate outcome measures to determine whether the treatment is not only safe but also efficacious. Vision outcomes used in clinical trials generally fall into two categories. First category of vision outcomes are designed to detect slowing of progression of disease. Examples of such outcomes include use of visual acuity, visual field and electroretinogram testing over many years in clinical trials of nutritional supplements [3, 4]. The second category of vision outcomes are designed to detect an improvement of vision occurring over a relatively short period following treatment. Examples of such outcomes include use of psychophysical methods to determine major increases in day and night vision following gene therapy [5, 6]. Usefulness of such improvements of vision to patients’ daily lives can be evaluated with mobility tests under carefully controlled laboratory conditions [7, 8].

Cone photoreceptor diseases (CPDs) are a subset of IRDs that preferentially affect day vision by causing impaired clarity of vision, abnormal color vision, unstable fixation, and reduced extent of visual fields; rod photoreceptor based night vision is either normal or relatively better preserved than day vision. CPDs include progressive cone and cone-rod dystrophies, and cone dysfunction syndromes that are classically described as stationary [9, 10]. The vision in CPDs is often seriously compromised by photoaversion, the high sensitivity to and discomfort from daytime ambient light levels [11]. Well known CPDs include achromatopsias caused by *CNGA3, CNGB3, GNAT2, PDE6C* and *PDE6H* gene mutations [12], blue-cone monochromacy caused by cone pigment mutations [13, 14], and cone-rod dystrophies caused by *ABCA4* mutations [15]. Less well known is the combination of CPD and congenital ultra-low vision that occurs in Leber congenital amaurosis (LCA) caused by *GUCY2D* mutations [16, 17]. Promising preclinical studies suggest *GUCY2D*-LCA could be treatable by gene therapy in order to improve the cone function [18, 19]. Efficacy outcome measures in clinical trials involving CPDs will likely include visual acuity, color vision, and fixation in order to detect improvements driven by cone vision as opposed to light-adapted rod vision. Also important would be orientation and mobility tests to determine the usefulness of cone vision improvements detected; the latter is especially important in the *GUCY2D*-LCA population with low vision.

We have previously published mobility performance metrics quantifying the ability of LCA patients to move through an indoor obstacle course at different ambient room illumination conditions [7]. It may be argued that a version of the same test limiting the conditions to the brightest illuminations could be applied to CPDs in order to measure day vision. However, our recent psychophysical studies have revealed that CPD patients adapt to their visual disability by using rod-based night vision function to accomplish many tasks that are normally performed with cone-based day vision [11, 14, 17]. Using mobility tasks in brighter ambient lights would not be helpful since CPD patients tend to squint to limit light-adaptation to their rod function. Here we describe a different strategy for evaluating cone-based vision for orientation in CPDs using a chromatic pseudo-natural scene in a controlled laboratory environment under free viewing conditions.

## Methods

### Subjects and clinical data

Patients (*n* = 7) with *GUCY2D-*LCA (Table 1) and subjects with healthy vision (*n* = 8) participated in this study. *GUCY2D*-LCA patients enrolled in the current study were different than those included in our previous publication [17]. All subjects had complete clinical ocular examinations, including best-corrected visual acuity. The tenets of the Declaration of Helsinki were followed, and informed consent and assent were obtained from all patients. The research was approved by the institutional review board at the University of Pennsylvania.

**Table 1:** Clinical and genetic characteristics of the *GUCY2D*-LCA patients

Patient, Gender	Age (y)	Allele 1	Allele 2	Eye	Visual acuity	Refractive error^a^
1 F	7	Ser448X	Ser448X	RE	HM	+3.25
				LE	20/800	+3.25
2 M	9	Arg768Trp	Arg768Trp	RE^b^	NLP	UP
				LE^b^	NLP	+6.00
3 F	11	Arg768Trp	Glu840 ins6bp	RE	20/250	−0.25
				LE	20/250	−2.00
4 M	12	Arg588Trp	Thr839 del1bp	RE	20/125	+2.75
				LE	20/125	+1.00
5 F	14	Arg768Trp	−146 T > C	RE	20/250	−2.50
				LE	20/400	−3.50
6 F	14	Gln156X	Ser819 del1bp	RE	LP	+5.00
				LE^b^	LP	UP
7 F	59	Thr312Met	Arg795Trp	RE	20/400	−0.50
				LE	20/400	−2.00

*F* female, *M* male, *RE* right eye, *LE* left eye, *LP* light perception, *HM* hand motion, *NLP* no light perception, *UP* unable to be performed

^a^Spherical equivalent

^b^Cataract

### Orientation device: discrimination of door on wall by rods and cones

**Fig. 1:**
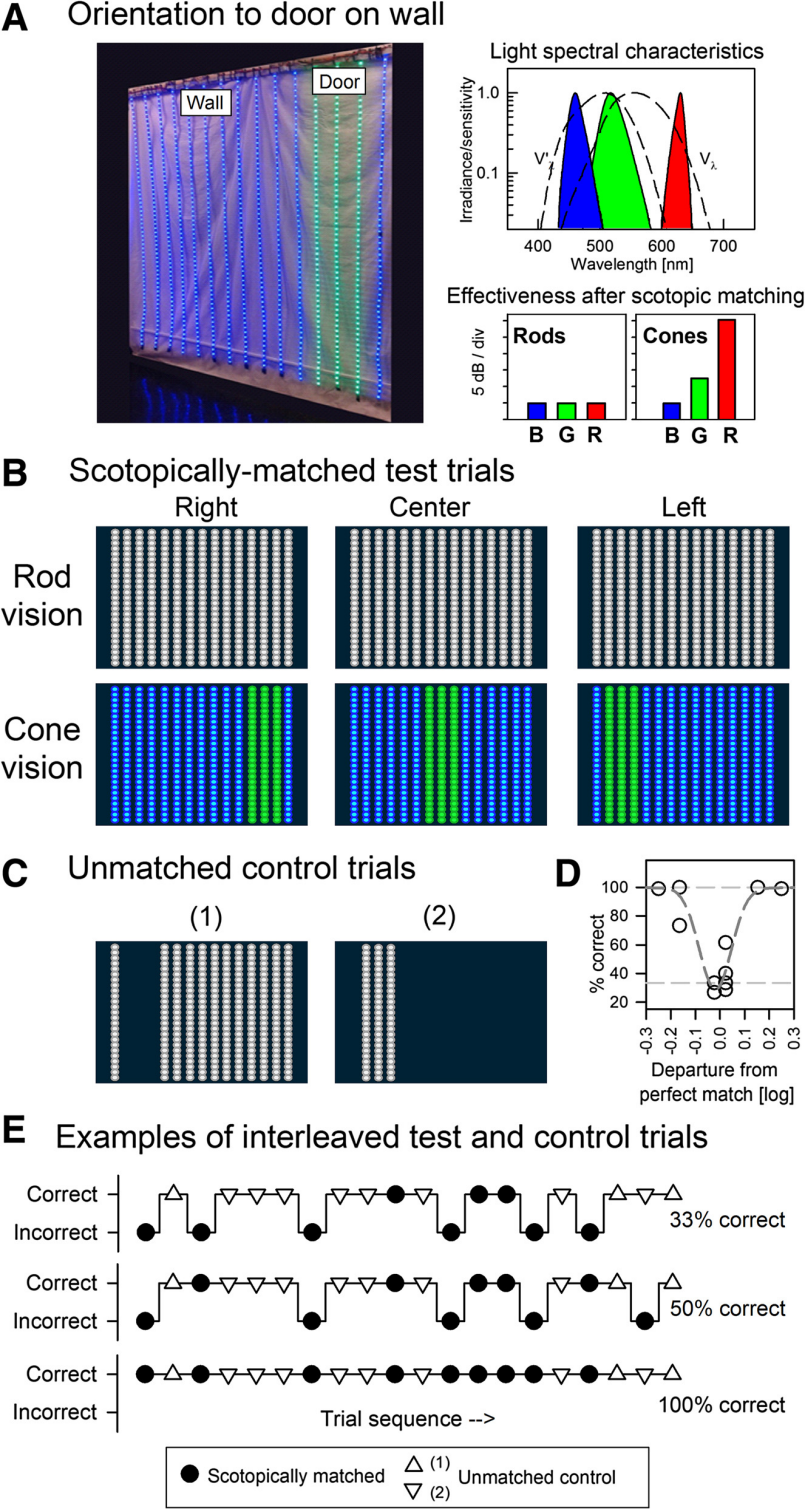
Presentation of scotopically matched scenes on an LED-lit wall. (**a**) Left, LED-lit wall and door device used for the three-alternative forced-choice design. A mix of light intensities for the “door” (three stripes at right, center or left) and “wall” (elsewhere) is calculated to be visible to cone-based vision but unable to be differentiated with rod-based scotopic vision. Right, spectral content of the LED lights compared to the spectral sensitivities of the rod (scotopic, V’_ʎ_) and cone (photopic, V_ʎ_) vision. All curves are normalized to unity at maximum. The relative intensities of the three lights are set such that they have the same effectiveness for rods (lower left) but substantially different effectiveness when perceived by cones (lower right). These differences permit differentiation of door and wall, and enhance visual orientation performance. (**b**) Rod vision is expected to perceive the scene as uniform stripes with no features (upper panels), and cone vision is expected to perceive the “door” as green stripes on a blue background (lower panels). (**c**) Appearance of unmatched control trials that should be visible to either rod or cone systems. (**d**) Departures of more than approximately 1.5 dB (0.15 log) from the scotopically matched mix of lights result in successful door-wall differentiation in normal subjects. (**e**) Representative examples of scotopically-matched test trials interleaved with unmatched control trials. For a three-alternative forced-choice setup, a subject with rod vision but no cone vision would be expected to get 33 % correct for matched-stimuli on average, whereas a subject using cone vision would be expected to get 100 % correct. Unmatched control presentations with door dimmer (1) or brighter (2) than wall are used to determine whether the subject has any vision (rod or cone or both) under the testing conditions

Fifteen tricolor LED strips (Neopixel RGB-PID1460, Adafruit Industries, NY) were hung with 0.1 m lateral separation to result in a vertical plane resembling a wall that is 1.4 m wide by 2 m high. The device subtends 34° horizontally and 48° vertically when observed from the test distance of 2 m. The LED strips were left free to flow vertically permitting traversal in a way similar to walking through a beaded curtain (Fig. 1a, left). The direction of the maximum emission of the LEDs was kept perpendicular to the plane by the montage to assure uniformity of light output across strips. Each vertical segment contained 60 triplets of individually addressable red, green and blue LEDs with peak wavelengths of 630, 518 and 460 nm, and FWHM of 14, 34 and 22 nm, respectively (Fig. 1a, upper right). LED spectra were measured with a spectrometer calibrated with a wavelength scale reference (USB2000 and Hg-1, Ocean Optics, Dunedin, FL). Each individual LED was controlled by a dedicated constant-current driver (WS2812B, WorldSemi, China) with 8-bit pulse-width-modulation (PWM) resolution permitting electronic luminance adjustment over a 2.41 log unit range. All strips were controlled using a single microcontroller (Arduino Mega, Ivrea, Italy) with an USB connection to a PC. Custom software was written for both the microcontroller (Wiring) and the PC application (Java, Linux). The device was programmed to produce a rectangular pattern target, 3-strip wide, defining the “door”, surrounded by the rest of the strips defining the “wall”. Two color combinations were used: green door on a blue wall (Fig. 1a, left), and a green door on a red wall (not shown). The perception of the door on the wall would be expected to depend on the luminance of each color and luminance difference between the colors. Also contributing to the perception are the adaptation state of the subject and the availability of functioning photoreceptor populations in the retina. For the main test trials, the intensities of the two lights used in each combination were chosen to be scotopically-matched and produced the same relative effectiveness for the rod-photoreceptor based vision. Due to the differences in the spectral sensitivity curves between scotopic and photopic systems, the scotopic-match would result in mismatched effectiveness for photopic cone-photoreceptor based vision (Fig. 1a, lower right). Specifically, scotopically matched combinations of blue/green and green/red produce a luminance contrast for cones of 0.8 and 2.5 log units, respectively (Fig. 1a, lower right). The scotopic matches were preliminarily determined using a radiometer calibrated for scotopic luminance (IL1700 with ZCIE filter, International Light, Peabody, Massachusetts) and the matches were fine-tuned near the normal rod absolute threshold based on results from dark-adapted normal observers using the final device implementation. Based on previous dark-adapted sensitivities obtained with two-color methods [17], we assumed scotopic matching in *GUCY2D*-LCA to be similar to normal subjects.

A three alternative forced-choice (3-AFC) experimental design was implemented by presenting the “door” at three locations (left, center or right) on the “wall” (Fig. 1b). The percent correct door position identification was estimated by conducting a set of at least ten trials, and required n ≥ 7 correct determinations to reject the null hypothesis of no discrimination at the α = 0.05 level, yielding a power of 88 %. Several sets of trials were performed sequentially to determine the percent correct as a function of scene luminance. Specifically, experiments started with fully-dark-adapted subjects and a very dim scene luminance, in which perception is rod-mediated in normal subjects, and it was increased until the dynamic range limit of the device was reached. The relative door/wall intensities were kept scotopically matched at all scene luminances. As full mobility course traversals for this number of trials would become prohibitive, we divided the assessment into two stages. A first stage was designed to determine the orientation capacity of subjects, and to obtain the contributions of rod and cone photoreceptor systems towards orientation capacity. The orientation sessions reported in the current work consisted of verbal assessments of the door location on the wall while the patient is located at the start of the course. The trials could proceed rapidly and total session durations were kept below 30 min. A second stage designed to obtain mobility-specific metrics and consisting of active course traversals may be developed in the future as treatment initiatives become available. Such a second mobility stage would piggyback on the orientation stage reported here by using a carefully selected subset of scene luminance parameters appropriate to each subjects’ level of vision. Also, the test paradigm is flexible enough to be adapted for use in the future in CPDs that differ in the relative losses of each cone subsystem, or in treatment approaches that may target preferentially one of the cone classes.

### Detailed methodology to determine orientation capacity

Each dark-adapted (>45 min.) subject is placed at the start of the course wearing a pair of goggles fitted with neutral density filters (Roscolux R98, Rosco Laboratories, Sun valley, CA). The goggles are used to adjust the scene luminance over a greater dynamic range than possible electronically, and do not interfere with the scene boundaries when looking straight ahead. The different levels of scene luminance are obtained by varying the number of neutral density filter sheets in the goggles. As the spectral absorbance of the individual sheets is not perfectly flat, the combined spectral characteristic of the stack of filters changes slightly as more sheets are added. To compensate for this effect, the resulting light output of the combination of device plus filter stack is kept scotopically matched at all times by small electronic adjustments of the LED PWM level.

A set of trials consists of no less than 10 scotopically-matched test trials plus a number of scotopically- and photopically-unmatched control trials (Fig. 1c, left). One of these sets is performed for each scene luminance, proceeding from dim to bright. The unmatched trials are used for control (should produce a success fraction close to 100 % for subjects having any form of orientation vision at the tested scene luminance) and to maintain orientation during the trials, and also to explain the test to subjects with very low vision. The contrast level for the control trials greatly exceeds the range of scotopic matches in normal subjects, which is approximately 0.4 log wide (Fig. 1d). Matched and mismatched trials are randomly interleaved by the software, with control (unmatched) trials being 20 % of the total trials on average. For each trial, a sound is produced after the adjustment of the wall and door intensities by the computer, and the subject is asked to verbally report the apparent door position as “Left”, “Center” or “Right”; they are encouraged to guess one of the three choices if unable to determine where the door is. The responses are recorded by the computer. Figure 1e illustrates representative examples of three sets producing different success rates (33, 50 and 100 %) for test trials (filled circles) with instances of interleaving control trial sequences (open triangles). Failure to discriminate the door position is signaled by a yield of ideally 33 % of correct answers to the 3-AFC design. Full discrimination implies near 100 % answers being correct. Results are acquired under free viewing conditions, except for illuminations requiring attenuating goggles.

All normal data were fit with a logistic function of the form

$$ p=\gamma +\frac{\left(1-\gamma -\lambda \right)}{\left(1+{e}^{\left(-\frac{\left(x-\theta \right)}{\sigma}\right)}\right)} $$

where *p* is the probability of correct, *x* is the scene luminance, *γ* is guessing probability (fixed to 0.3) and *λ* is the lapsing probability (fixed to 0) [20]. The spread parameter, σ, was allowed to vary for normal results but held constant at the normal value for the patient results. The threshold parameter, *θ,* was allowed to vary for both normals and patients.

## Results

### Normal subjects

**Fig. 2:**
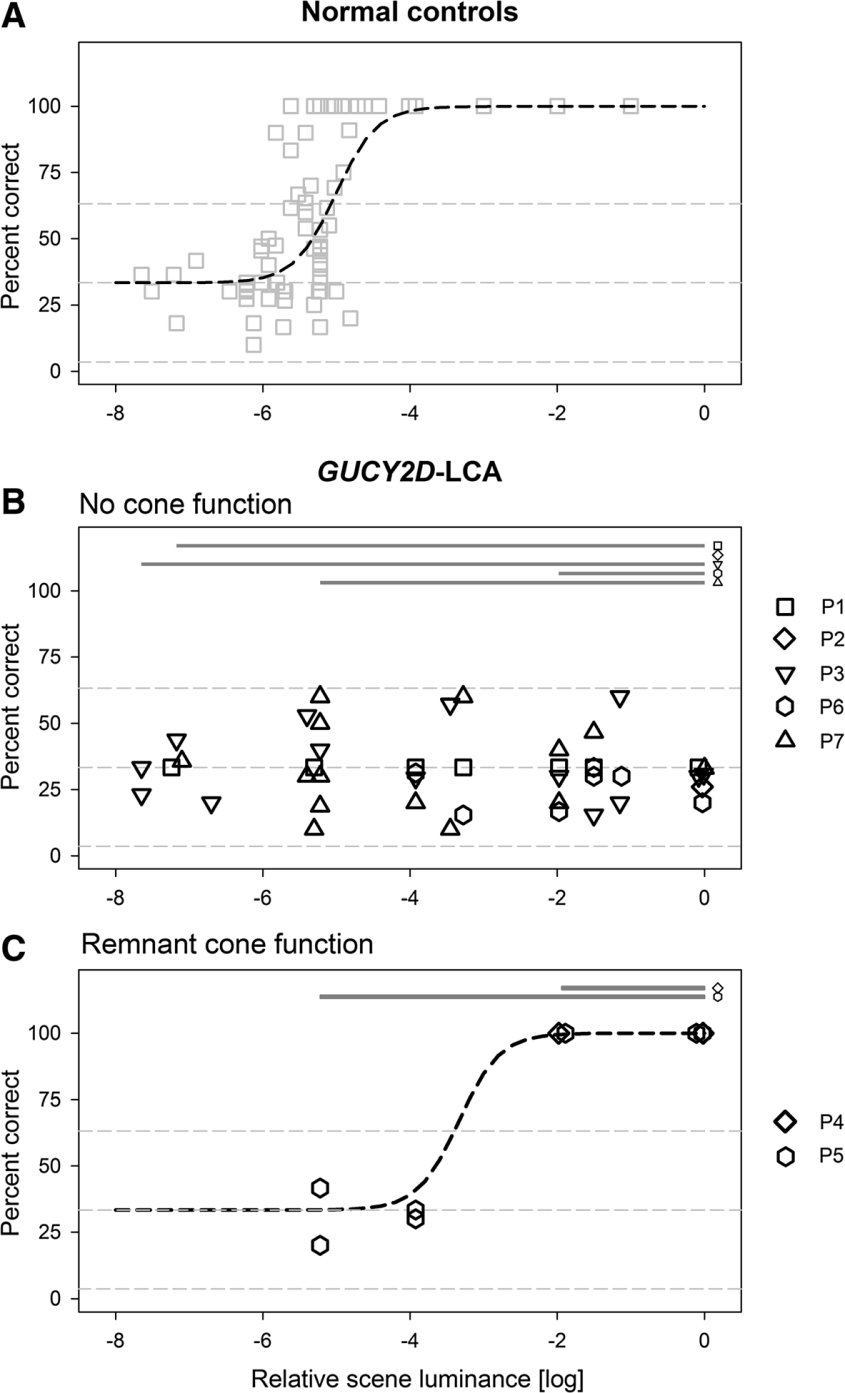
Identification of cone vision as a function of scene luminance. (**a**) Percent correct identification of the location of photopically-mismatched but scotopically-matched trials using a three-alternative forced-choice method in normals as a function of scene luminance. Near normal scotopic threshold (7–8 log attenuation), subjects cannot differentiate between door and wall any better than chance. Upon increase in scene luminance, colors become visible and door identification accuracy reaches 100 %. The sigmoidal curve is a logistic function fitted to the data, with parameters θ = −5.01 log (threshold) and σ = 0.28 log (spread). (**b**) Five *GUCY2D*-LCA patients that fail to perform the visual orientation task with scotopically-matched trials across the full 8 log unit dynamic range of scene luminances (symbols). Four of the patients successfully orient with unmatched control trials (upper gray bars) over a range of scene luminances suggesting their use of scotopic vision. (**c**) Two *GUCY2D*-LCA patients who can correctly perform visual orientation task with scotopically-matched and unmatched trials but require greater scene luminance than normal. Fitted function and parameters as in (A) except for the shifted threshold parameter, which resulted in θ = −3.3 log. Both green/blue and green/red data are shown

The dimmest scene luminance (8 log attenuation from maximum) used in the current work was well within the range of normal scotopic vision. With unmatched control trials, normal subjects reliably (>65 %) saw the location of the door on the wall but did not report perception of color (not shown). For the scotopically-matched test trials, door and wall were unable to be differentiated by normal subjects. The average percent correct (identification success rate) was within the range of hypothetical probability given just by chance (mean 33 %; 95 % confidence interval 4 %-63 %; *n* = 10 trials; binomial), as expected from random guessing for a 3-AFC design (Fig. 2a). Similar results were obtained for a green door on a blue wall, and for a green door on a red wall. When the scene luminance was increased by approximately 2 log units (~6 log attenuation) success rate remained near chance (33 %) suggesting the continued lack of visibility by the normal photopic cone system. Upon a further increase of the scene luminance by an additional 2 log units (~4 log attenuation), there was a clear increase in percent correct, indicating the onset of normal cone vision occurring between 6 and 4 log attenuation with the threshold (*θ*) being on average at 5 log attenuation (Fig. 2a). At ~4 log attenuation and higher levels, subjects also reported the color of the wall and door for both scotopically-matched test trials and unmatched control trials. This perceptual behavior continued through the maximum available scene luminance of 0 log attenuation (Fig. 2a).

### *GUCY2D*-LCA patients with no evidence of cone function

We first considered a subset of four *GUCY2D*-LCA patients (P1, P3, P6, and P7) with visual acuities ranging from light perception (LP) to 20/250 (Table 1). With the newly developed orientation task, P1 and P3 had no difficulty correctly identifying the location of the door on the wall during unmatched control trials performed with dim scene luminances between 8 and 6 log attenuation (Fig. 2b, upper gray bars). These results were consistent with their relatively retained rod vision determined psychophysically. Throughout the rest of the brighter scene luminances, these two patients continued to correctly identify the location of the door on unmatched control trials (Fig. 2b, upper gray bars) but performed no different than pure guessing for the scotopically-matched test trials (Fig. 2b, symbols). P6 and P7 required higher scene luminances, approximately 5 and 2 log attenuation, respectively, in order to correctly identify the location of the door during unmatched control trials (Fig. 2b, upper gray bars). Again throughout the rest of the brighter scene luminances, P6 and P7 performed correctly on unmatched control trials but not on scotopically-matched test trials (Fig. 2b, symbols). The results P1, P3, P6 and P7 were consistent with no cone-based vision being used for the orientation task and retained rod-based vision functioning adequately for orientation even under bright scene luminance conditions [17]. Last to be considered was P2 who could not perceive any light and he was included for method validation. Appropriate for the lack of light perception, P2’s orientation results were no different than guessing for both unmatched control and scotopically-matched test trials. His results were consistent with lack of rod- and cone-based vision over the full 8 log unit dynamic range tested.

### *GUCY2D*-LCA1 patients with detectable but abnormal cone function

Next we considered two *GUCY2D*-LCA patients (P4 and P5) in whom visual acuities ranged from 20/125 to 20/400 (Table 1). With unmatched control trials, P5 could identify the location of the door near 5 log attenuation and at higher scene luminances (Fig. 2c, upper gray bars). With scotopically-matched test trials, at approximately 2 log attenuation P5 could successfully identify the location with nearly 100 % accuracy. The capacity to visually orient to both unmatched as well as matched trials continued to the brightest scene luminance. P4 on the other hand, did not identify correctly either matched or unmatched trials throughout the range up to the 2 log attenuation. At that luminance and maximum luminance, P4 could correctly identify both matched and unmatched trials. These results suggested a detectable cone based vision in P4 and P5 with a threshold between 2 and 4 log attenuation (average threshold *θ* = 3.3 log) which would correspond to an approximate 2 log elevation compared to normal cone vision. P5 had better scotopic vision compared to P4.

## Discussion

Evaluation of the real-world consequences of incremental improvements achieved by novel treatment strategies is very challenging in clinical trials of patients with ultra-low vision. Orientation and mobility (O&M) capacity can be evaluated either by observation of patients tending to natural tasks in the real-world [21], or measurement of their performance in courses built within controlled environments under standardized conditions that simulate some aspects of the real-world [7, 8, 22], or in virtual reality environments [23, 24]. In the current context, orientation can be defined as a process by which position is established using visual cues with respect to features and obstacles within the environment; non-visual orientation, such as use of echolocation [25] is excluded. Mobility, on the other hand, is defined as the capacity of the subject to use the mental map of the environment created by a preceding orientation step to reach a pre-specified feature avoiding any obstacles that may be on the path [26]. Thus evaluation of orientation can be thought of as a prerequisite to the evaluation of vision-based mobility performance (as opposed to non-visual assistive tools such as a cane). O&M performance strongly depends on visual field extent, scotoma location, and visual acuity [27–29]. One of the important considerations for O&M evaluation is the control of ambient light environment and the apparent contrast of features therein [30]. For example, *RPE65*-LCA patients behave like sighted individuals in a brightly lit mobility course but behave as severely visually impaired in a dim light environment [7].

*GUCY2D*-LCA is one of the more common molecular causes of LCA and involves the phototransduction pathway of both rod and cone photoreceptors [17, 31–33]. There is usually severe vision loss with low visual acuity and profound photoreceptor dysfunction by retinal electrophysiology [16, 17, 32]. *GUCY2D*-LCA is unlike any other IRD described to date because it invariably demonstrates a retinal laminar structure that is near normal and completely dissociated from the extremely severe visual impairment experienced by the patients. Using full-field psychophysical methods, cone sensitivity losses tend to be much greater than rod sensitivity losses [17] making *GUCY2D*-LCA a form of CPD often associated with congenital ultra-low vision.

As a first step in evaluating O&M performance in *GUCY2D*-LCA patients, we designed and built an orientation device consisting of a “door” on a “wall”. We used chromatic contrast as a visual cue in order to avoid use of the rod-photoreceptor based night vision of patients for day vision tasks such as it occurs in other CPD patients [11, 14]. Previous investigators have stressed the importance of using relatively short sessions on a simple course design of reduced dimensions and repeated measurements to assess severely affected patients in a reasonable time [34]. It was concluded that learning effects are unlikely to confound assessment in those conditions. We pursued similar goals by utilizing a device with a reasonably wide coverage of field of view under free viewing conditions and a rapid way of obtaining replications by using the patient’s verbally reported door localization information.

## Conclusions

Novel treatment trials such as gene augmentation therapy are in the planning stages for CPDs. Here we developed a device and a technique which can be used as an outcome measure to evaluate the cone-vision-based orientation capacity of CPD patients undergoing treatment trials.

## Abbreviations

FWHM: Full-width at half-maximum
GUCY2D: Guanylate cyclase 2D, membrane (retina-specific)
LCA: Leber congenital amaurosis
LED: Light emitting diode
RPE65: Retinal pigment epithelium-specific protein 65 kDa
PWM: Pulse-width modulation
